# The Influence of Quinine in Parturition

**Published:** 1876-12

**Authors:** D. P. Duncan


### THE INFLUENCE OF QUININE IN PARTURITION.

BY D. P. DUNCAN, M.D.

So many articles have been written upon this subject that I have forborne saying anything, anticipating a successful refutal of the doctrines maintained by those who endorse its "so-called" action upon the uterus.

That it possesses no such property, I am convinced, and feel assured that all thinking men of the profession of from fifteen to twenty years' experience, will agree with me when they recount the numberless cases where quinine has been used in heroic doses to combat chill in women who were in an "enciente" condition.

Quinine is a powerful tonic, and no doubt in this way may induce labor when the system needs a tonic to perfect normal deliveries. Or in the case of an anæmic patient, where the full term was nearly reached. Or where excitation in a peculiarly sensitive female, I doubt not but that it might produce it by its action as a tonic and stimulant, as any other tonic or stimulant might do. But, as to its possessing any specific action under these circumstances, I conceive simply absurd. When we learn to reflect more and write less, we may expect medical literature to improve in tone and accuracy—and then we shall be relieved of the intolerable burthen of being forced to read an octavo volume to gain a fact.

We do not appreciate the exercise of talent in the elaborate discussion of the action of medicines in any individual case; but we need the experience of sound thinkers in the treatment of a dozen or more cases with similar treatment in each. When successful in the above number, we then can safely conclude that we have substantiated a theory, and drink it in as a truth from the fount of Knowledge.

This worthless literature is food for the charlatan, who, for "filthy lucre," has launched his boat upon the broad ocean of philanthropy without the rudder of science to sustain or direct him. When he fails to relieve suffering humanity, he appeals to the literature of medical journalism, and is sustained by the hundred and one different remedies there offered in the treatment of special cases, and gains strong support for the continuance of his reckless and unscientific course.

Let us be guarded in allowing space to the experience or observations upon reputed cures in individual cases, and *then*, and not till then, may we expect the physician to accomplish the noble object, which it is presumed the title of Doctor of Medicine confers upon him the power to do.

We do not pretend to heal by miraculous interposition; but the "Great Master" has spread before us the vegetable and mineral kingdoms, from whose vast and inexhaustable resources we are to gather balm for the afflicted, and in the application of any of these to the treatment of disease, we should feel confident that we have established a fact, before we attempt to promulgate it.

